# A comprehensive review on recent nanosystems for enhancing antifungal activity of fenticonazole nitrate from different routes of administration

**DOI:** 10.1080/10717544.2023.2179129

**Published:** 2023-02-14

**Authors:** Sadek Ahmed, Maha M. Amin, Sinar Sayed

**Affiliations:** Department of Pharmaceutics and Industrial Pharmacy, Faculty of Pharmacy, Cairo University, Cairo, Egypt

**Keywords:** Fenticonazole Nitrate, terpesomes, olaminosomes, confidence interval and docking study

## Abstract

This review aims to comprehensively highlight the recent nanosystems enclosing Fenticonazole nitrate (FTN) and to compare between them regarding preparation techniques, studied factors and responses. Moreover, the optimum formulae were compared in terms of *in vitro, ex vivo* and *in vivo* studies in order to detect the best formula. FTN is a potent antifungal imidazole compound that had been used for treatment of many dangerous fungal infections affecting eye, skin or vagina. FTN had been incorporated in various innovative nanosystems in the recent years in order to achieve significant recovery such as olaminosomes, novasomes, cerosomes, terpesomes and trans-novasomes. These nanosystems were formulated by various techniques (ethanol injection or thin film hydration) utilizing different statistical designs (Box-Behnken, central composite, full factorial and D-optimal). Different factors were studied in each nanosystem regarding its composition as surfactant concentrations, surfactant type, amount of oleic acid, cholesterol, oleylamine, ceramide, sodium deoxycholate, terpene concentration and ethanol concentration. Numerous responses were studied such as percent entrapment efficiency (EE%), particle size (PS), poly-dispersity index (PDI), zeta potential (ZP), and *in vitro* drug release. Selection of the optimum formula was based on numerical optimization accomplished by Design-Expert® software taking in consideration the largest EE %, ZP (as absolute value) and *in vitro* drug release and lowest PS and PDI. *In vitro* comparisons were done employing different techniques such as Transmission electron microscopy, pH determination, effect of gamma sterilization, elasticity evaluation and docking study. In addition to, *ex vivo* permeation, *in vivo* irritancy test, histopathological, antifungal activity and Kinetic study.

## 1. Introduction

Although many fungi exist in our regular life without any harmful effect, the prevalence of fungal infections seems to be increasing in the global world. Under specific conditions, some fungi could result in medical conditions that vary from mild to life threating infections. The leading risk factors include modulation of the immune system that happens as a result of viral infection, anti-cancer medications and transplantation surgeries (Lockhart & Guarner, 2019). Fungal infection could affect different body regions such as the eye, the skin and the vagina, thereby these infections could be detected by taking samples from blood, urine, sputum or vaginal sections. Fungal keratitis is an ocular infection that happens only in traumatic cornea and caused by many *Candida* species (Goldschmidt et al., 2012). *Trichophyton* species and *Candida albicans* trigger many skin infections (Albash et al., 2021). For gynecologists, fungal infections caused by *Candida albicans* represent the main cause of women upset and may result in premature birth or even abortion, pelvic inflammation and transmission of sexual diseases (Martinez-Perez et al., 2018).

There are many antifungal drugs such as azoles, polyenes and allylamine with different mechanism of actions. Azoles have a dual action as it acts as fungistatic at low concentration as they interfere the ergosterol formation, and fungicidal at high concentration resulted from the complete destruction of cell wall (Chaudhari et al., 2022). Fenticonazole Nitrate (FTN) is a potent azole compound with a well-recognized antifungal activity, thereby used to treat fungal infections affecting different body parts. The aqueous solubility of FTN represents its main drawback as it is less than 0.10 mg/mL. Poor aqueous solubility could significantly affect the drug activity or even results in microbial resistance, thereby novel nanosystems had been developed (Ahmed et al., 2022).

Nanosystems production faces many challenges as we need to obtain both acceptable safety and efficacy. Accordingly, various fabrication techniques and characterization processes had been adopted (Sondi & Salopek-Sondi, 2004). It’s important to note that characterization techniques start from the early stages of developments in terms of EE %, PS, ZP, PDI and drug release to select the best possible combination of components using a remarkable statistical designs and software. Thereby, different factors such as surfactant concentrations, surfactant type, amount of oleic acid, stearic acid, cholesterol, oleylamine, ceramide, sodium deoxycholate, terpene concentration, terpene type and ethanol concentration had been adopted. After that, the optimum formulae were subjected to extensive *in vitro* characterization, *ex vivo* and *in vivo* studies to validate their safety and efficacy.

They are many statistical designs that could be used in order to optimize the fabrication process such as central composite, Box-Behnken, full factorial and D-optimal designs. These designs belong to response surface methodology (RSM) techniques that provide several equations that facilitate the detection of the optimum formula according to the required criteria (largest EE %, ZP as absolute value and *in vitro* drug release and lowest PS and PDI) (Ahmed et al., 2021). Confidence interval is extremely important to detect the significant differences between the studied formulae. As a general rule, a non-significant (*p* > 0.05) difference is expected when the confidence intervals of the studied formulae intersect, otherwise a significant difference exists (*p* < 0.05). Precisely the goal of this review is to clearly highpoint the latest nanocarriers of FTN and to compare between them from the early fabrication and selection of statistical model moving to the investigated factors and responses. Afterward, the optimum formulae were matched comprehensively regarding the different *in vitro, ex vivo* and *in vivo* studies aiming to expect the best formula.

## 2. Innovative nanosystems

This review compare between the recently used nanosystems entrapping FTN. They could be classified in this review according to the targeted site of action into ocular, topical or vaginal. Ocular nanosystems include olaminosomes (O-OLN), novasomes (O-NV) and terpesomes (O-TP) (Albash et al., 2021; Ahmed et al., 2022a,b). Topical nanosystems resemble trans-novasomes (T-T-NV) and cerosomes (T-CE) (Albash et al., 2021; 2022). Vaginal nanosystems include sodium deoxycholate (SDC) and ethanol-containing terpesomes (V-TP) (Albash et al., 2020). The composition of each formula controls its properties, safety and activity. Changing the amount of any component could significantly affect the desired outcomes, thereby the effects of different factors on different responses are discussed comprehensively.

Regarding ocular nanosystems, olaminosomes represent novel nanocarriers that are chiefly composed of oleic acid, oleylamine and surfactant (Abd-Elsalam & ElKasabgy, 2019). Oleic acid is an unsaturated free fatty acid that is widely used in nanosystems formulation as a result of its safety, biodegradability and biocompatibility. Oleylamine is a long-chain amino compound with pronounced capping properties that extremely affect the stability, permeability and activity (Safo et al., 2019). Surfactants are extensively used in nanosystems as they optimize their surface and penetration properties (Abd-Elsalam & Ibrahim, 2021). Span 80 was used in O-OLN and O-NV.

Novasomes are enhanced niosomal structure that is chiefly composed of stearic acid, cholesterol and surfactant. Stearic acid is a well-recognized fatty acid with high safety properties (Singh et al., 2020). Cholesterol is a naturally produced compound that control plasma membrane rigidity, formation of bile acid and steroids. In formulation industry, it had an important role in controlling vesicles EE%, permeability and stability (Abdelbary et al., 2017). Finally, terpesomes are mainly composed of terpenes and phospholipid. Terpenes like limonene, fenchone and eugenol are derived from essential oil and had both antifungal and penetration enhancers properties (Younes et al., 2018). Phospholipids are assembled together with cholesterol to form the plasma membrane. They regulate many cellular processes such as membrane permeability, cell growth and apotheosis (Morita & Ikeda, 2022).

Considering the topical nanosystems, cerosomes are composed of ceramide, phospholipid and surfactants. Ceramides are simple hydrophobic component that represent 50% of stratum corneum, thereby extensively used in long-term skin protection products. Their presence could optimize vesicle particle size, EE %, ZP and permeation (Su et al., 2017). However, Trans-novasomes represent a modification of novasomes in order to augment its topical activity against tinea corporis. They are composed of oleic acid (instead of stearic acid), cholesterol, span 60 (instead of span 80) and Brij (additional surfactant). The polyethylene glycol (PEG) content of Brij has the ability to enhance topical permeation into deep tissues and to extend the retention time (Vega et al., 2013; Rangsimawong et al., 2014).

Regarding the vaginal nanosystems, V-TP composition had some extra components in addition to the terpenes which were SDC and ethanol. SDC is a bile salt that demonstrated a pronounced effects regarding EE %, PS, ZP and permeability (Ahmed et al., 2020). Moreover, ethanol has a fluidizing effect that could improve permeation.

## 3. Statistical designs

In order to study the effect and interaction of different factors, employing statistical designs are highly recommended. They also facilitate the selection of the optimum formula by numerical optimization that is based on a response surface methodology (RSM) (Ahmed et al., 2021). In our study, various statistical designs were employed such as central composite, Box-Behnken, full factorial and D-optimal designs. Ocular novasomes (O-NV) and olaminosomes (O-OLN) were constructed using central composite design which was represented by twenty trials and Alpha of 1.68179 (Ahmed et al., 2022a,b). However, ocular terpesomes (O-TP) and topical cerosomes (T-CE) were formulated utilizing full factorial designs (Albash et al., 2021a,b). Moreover, vaginal terpesomes (V-TP) were prepared adopting a Box-Behnken design (Albash et al., 2020). Finally, D-optimal design was adopted to formulate topical trans-novasomes (T-T-NV) (Albash et al., 2022). All the previous designs require few points, but the central composite designs offer elevated exactness, reasonable quantity of data for evaluating the appropriateness of fit in comparison with the three level full factorial design (Imanian & Biglari, 2022). The studied factors and measured responses of the formulated nanosystems are represented in Table 1. In order to detect the significance of these factors, Design-Expert^®^ software was used.

**Table 1.** Route, nanosystems, statistical design, preparation technique, studied factors and measured responses of the different FTN-loaded nanosystems.

Route	Topical		Ocular			Vaginal
Nanosystem	T-T-NV	T-CE	O-NV	O-OLN	O-TP	V-TP
statistical Design	D-optimal	Full Factorial Design	Central composite	Central composite	Full Factorial Design	Box-Behnken
Preparation technique	ethanol injection	Thin film	ethanol injection	ethanol injection	Thin film	Thin film
Studied Factors	Span 60 amount oleic acid amount Brij type	ceramide amount Brij type Brij amount	stearic acid concentration span 80: drug ratio cholesterol amount	span 80 amount oleylamine concentration oleic acid: drug ratio	terpenes type terpenes amount	terpenes ratio SDC amount ethanol concentration
Measured Responses	EE % PS PDI ZP	EE % PS PDI ZP	EE % PS PDI ZP Release (8h)	EE % PS PDI ZP Release (10h)	EE % PS PDI	EE % PS PDI

**Abbreviations:** EE %, percent entrapment efficiency; PDI, poly-dispersity index; PS, particle size; ZP, zeta potential.

**Nanosystems:** O-NV, Ocular Novasomes; O-OLN, Ocular Olaminosomes; O-TP, Ocular Terpesomes; T-CE, Topical cerosomes; T-T-NV, Topical Trans-Novasomes; V-TP, Vaginal Terpesomes.

The optimum formulae of the six nanosystems were compared using the determined confidence Intervals. Confidence interval (CI) represents a range of values where we believe that our true value will lie. The confidence level equals (1-significance level). For our statistical studies the significance level (alpha) would be 0.05, so the CI would be 95%. This interval represents the most commonly used interval in statistical studies and it could be calculated from the following equation:

(Eq. 1) $$95\%\;CI=\text{ Ẋ }\pm t\;\frac{s}{\sqrt{n}}$$

Ẋ, t, s and n are the sample mean, t-score, sample standard deviation and sample size respectively. T-score is a constant related to degree of freedom and significance level and is used for small sample sizes. Generally, a significant difference (*p* < 0.05) exists when the point lies outside the CI, however a non-significant difference (*p* > 0.05) is expected when the point lies inside the CI. Since each studied variable had its own mean (Ẋ) and standard deviation (s). A non-significant (*p* > 0.05) difference is expected when the confidence intervals intersect, otherwise a significant difference exists (*p* < 0.05).

## 4. Preparation techniques

Different nanosystems were formulated utilizing either ethanol injection or thin film hydration techniques. In ethanol injection method, FTN and other components were dissolved in ethanol at 60 °C. Then the resulted solution was injected dropwise into larger-volume of phosphate-buffered saline (PBS, pH 7.4) (Ahmed et al., 2022a,b) or distilled water and magnetically stirred at the same temperature till disappearance of alcohol (Albash et al., 2022). The final product was sonicated to reduce the PS then stored at 4 °C (Al-Mahallawi et al., 2014). However, in thin film hydration technique FTN and the additional constituents were dissolved in methanol (Albash et al., 2020; 2021) or chloroform: methanol (2:1 V/V) mixture (Albash et al., 2021). The organic solvent was then slowly evaporated at 60 °C and 90 rpm utilizing rotor evaporator. The resulted thin film was then hydrated with distilled water (Albash et al., 2020; 2021), sonicated then stored at 4 °C overnight to mature the vesicles.

## 5. Studied responses and factors

### 5.1. Studied responses

In order to characterize the resulted formula, different *in vitro* responses were measured. These responses were divided into percent entrapment efficiency (EE%), particle size (PS), poly-dispersity index (PDI), zeta potential (ZP), and *in vitro* drug release. EE % could be determined spectrophotometrically either directly or indirectly. Briefly, 1 mL of resulted formula is subjected to cooling centrifugation at 21,000 rpm for 1 hour at 4 °C. If the supernatant was diluted, then EE % would be measured indirectly utilizing equation 2 (Ahmed et al., 2020; Sayed et al., 2020). However, if the sediment was lysed using methanol then EE % would be measured directly as represented in equation 3 (Abdellatif et al., 2017). Total amount of FTN is the actual amount used, total amount of free FTN (quantity of FTN in supernatant), total amount of entrapped FTN (quantity of FTN in sediment). EE % was determined directly for O-TP, T-CE, T-T-NV and V-TP. Moreover, indirectly for O-NV and O-OLN. EE % is an important response, since the efficacy of the nanosystem is directly proportional to its EE % (Abdelbary et al., 2016).

 (Eq. 2) $$EE\;\;\%\;\;=\frac{\left(\text{total}\;\;\text{amount}\;\;\text{of}\;\;\text{FTN}-\;\;\text{total}\;\;\text{amount}\;\;\text{of}\;\;\text{free}\;\;\text{FTN}\right)}{\text{total}\;\;\text{amount}\;\;\text{of}\;\;\text{FTN}}\;\;\text{X}\;\;\;100$$

(Eq. 3) $$EE\;\;\%\;\;=\;\;\frac{\text{Entrapped}\;\;\text{amount}\;\;\text{of}\;\;\text{FTN}}{\text{total}\;\;\text{amount}\;\;\text{of}\;\;\text{FTN}}\;\;\text{X}\;\;\;100$$

Zetasizer was used to determine PS, ZP and PDI of the resulted formulae after a suitable dilution (Abd-Elsalam & ElKasabgy, 2019). Small PS is of a high importance to ensure effective permeation and tolerance with the targeted region (Younes et al., 2018). ZP gives high indication of the physical stability as it directly affects the possible interaction between the adjacent particles and prevents aggregation (Albash et al., 2021). The homogeneity of the resulted formula was detected through PDI. The closed the PDI to zero, the higher the homogeneity of the system (Mosallam et al., 2021).

The last *in vitro* response was drug release. *In vitro* drug release was studied only in O-NV and O-OLN adopting bag dialysis technique (typical molecular weight cutoff 14,000 Da; Sigma-Aldrich Co.) (Elsayed & Sayed, 2017). During this method several aliquots were collected in order to determine the progress in drug release. After that the release profiles were fitted to zero, first, and Higuchi diffusion models (Ahmed et al., 2020).

### 5.2. Studied factors

The effects of studied independent variables of each nanosystem on the measured responses are briefly illustrated in Table 2.

**Table 2.** Effect of different factors on studied responses.

Factor	↑ Surfactant amount				↑ Oleic acid amount		Surfactant type		↑Cholesterol amount
Nanosystem	O-NV	O-OLN	T-T-NV	T-CE	O-OLN	T-T-NV	T-T-NV	T-CE	O-NV
EE %	+	+	+	–	+	–	+ (more lipophilic)	+ ( more lipophilic)	+
PS	+	–	+	–	–	+	+ (lower PEG content)	+ ( lower PEG content)	+
ZD	+	ns	ns	ns	+	ns	+ (more lipophilic)	+ (more lipophilic)	+
PDI	ns	ns	ns	ns	ns	+	ns	ns	ns
Release	ns	+	not studied	not studied	ns	not studied	not studied	not studied	–
Factor	**↑ stearic acid amount**	**↑ ceramide amount**	**↑ oleylamine amount**	**↑ Terpenes amount**		**terpenes type**	**↑ SDC amount**	**↑ ethanol concentration**	
Nanosystem	O-NV	T-CE	O-OLN	O-TP	V-TP	O-TP	V-TP	V-TP	
EE %	+	+	+	–	–	+ (more lipophilic)	–	–	
PS	+	+	ns	–	–	+ ( more lipophilic)	–	–	
ZD	+	–	+	not studied	ns	not studied	ns	ns	
PDI	ns	ns	ns	–	ns	ns	ns	ns	
Release	–	not studied	+	not studied	not studied	not studied	not studied	not studied	

**Abbreviations:** EE %, percent entrapment efficiency; PDI, poly-dispersity index; PEG, polyethylene glycol; PS, particle size; ZP, zeta potential.

**Significance:** +, Positive significant effect; -, negative significant effect; ns, non-significant effect.

**Nanosystems:** O-NV, Ocular Novasomes; O-OLN, Ocular Olaminosomes; O-TP, Ocular Terpesomes; T-CE, Topical cerosomes; T-T-NV, Topical Trans-Novasomes; V-TP, Vaginal Terpesomes.

#### 5.2.1. Surfactant amount

Span 80 was incorporated in the formation of O-NV and O-OLN (Ahmed et al., 2022a,b), however Brij was used during the construction of T-CE (Albash et al., 2021). Both Brij and span 60 were employed in the formation of T-T-NV (Albash et al., 2022). Regarding EE %, increasing the amount of surfactant had a positive significant effect in O-NV, O-OLN and T-T-NV, but it showed a negative significant effect in T-CE. Positive effect could result from the reduced fluidity of bilayers due to the large alkyl chain moiety that lead to low HLB (Abdelbari et al., 2021). Moreover, increasing the stabilization, emulsification effect, lipophilicity of the medium (Mosallam et al., 2021). Finally, more lipophilic vesicles would be formed as the concentration of surfactants increase (El-Laithy et al., 2011). In T-CE the incorporation of large amount of surfactants with high HLB value, unsaturated double bond and acyl group lead to formation of leaky bilayers, hence EE % decreases (Al-Mahallawi et al., 2014).

Regarding PS, positive significant effect in O-NV and T-T-NV could be explained in terms of the large alkyl chain of span that resulted in large core space, hence allowing the entrapment of more FTN and increasing the PS (Hathout et al., 2007). Negative significant effect in O-OLN and T-CE could be clarified in terms of increasing the surface coverage that reduces the interfacial tension and aggregation of adjacent particles (Eldeeb Salah & Ghorab, 2019). Considering ZP, only O-NV showed a positive significant effect that resulted from the free ionizable hydroxyl group (Ahmed et al., 2022). O-OLN was the only formula that revealed a positive significant effect on release as a result of the improved solubility of FTN (Abd-Elsalam & Ibrahim, 2021). Surfactant amount showed a non-significant effect on PDI in all studied formulae.

#### 5.2.2. Surfactant type

Changing the surfactant type had been accomplished in T-T-NV (Brij 93 and Brij 58) and T-CE (Brij 97 and Brij 52) (Albash et al., 2021; 2022). HLB of Brij 93, Brij 58, Brij 97 and Brij 52 are (4,15.7, 12.4 and 5.3 respectively) (Tagami et al., 2011; Mosallam et al., 2021). As a general rule, The lower the HLB value, the longer the alkyl chain that increases the lipophilicity of surfactant as a result of formation of less hydrophilic holes in the bilayer, hence diminishing its fluidity (Abdelbari et al., 2021). Regarding EE %, higher EE % was noticed in both T-T-NV and T-CE upon incorporation of the lower HLB surfactant. Considering PS, surfactant with lower polyethylene glycol (PEG) content would have a larger PS as a result of agglomeration of the vesicles (Caliceti et al., 2004). PEG content of Brij 93, Brij 58, Brij 97 and Brij 52 are (2, 20, 10 and 2 respectively). Moreover, the surfactants with lower PEG content are more lipophilic (lower HLB value), hence more FTN would be entrapped and the PS would increase (Hathout et al., 2007; Ahmed et al., 2020). Regarding ZP, surfactants with higher PEG content would adhere to the surface of nanosystem, hence reducing the charge on the surface as a result of stearic hindrance (Aziz et al., 2018). Both T-T-NV and T-CE showed higher ZP upon incorporation of the more lipophilic surfactant (lower HLB, lower PEG content). Changing surfactant type revealed a non-significant effect on PDI.

#### 5.2.3. Oleic acid amount

Changing the amount of oleic acid was observed in O-OLN and T-T-NV (Ahmed et al., 2022; Albash et al., 2022). O-OLN showed a positive significant effect on EE % upon increasing the amount of oleic acid which could be explained in terms of lipophilicity since oleic acid is a long chain fatty acid (C_18_H_34_O_2_) (Mishra et al., 2022). Moreover, presence of more oleic acid would allow efficient interaction with the capping agent oleylamine and formation of amide bond (Safo et al., 2019). However, T-T-NV revealed a negative significant effect on EE % as a result of destruction of lipid matrix by the excessive oleic acid (Zhang et al., 2013). Regarding PS, O-OLN demonstrated a negative significant effect as a result of reduction of surface tension and rigidity of the bilayer resulted from its penetration enhancing properties, thus reducing PS (Song et al., 2012). However, T-T-NV showed a positive significant effect resulted from the stearic attractive force with oil molecules plus the angular structure of oleic acid, thus PS increases (Shukla et al., 2003; Sarheed et al., 2020). Considering ZP, only O-OLN showed a positive significant effect resulted from the presence of ionizable carboxylic group (Abd-Elsalam & ElKasabgy, 2019). Regarding PDI, T-T-NV demonstrated a positive significant effect due to the direct association between PS and PDI. The larger the PS the more the PDI (Kumar et al., 2014). Changing the amount of oleic acid revealed a non-significant effect on release (Ahmed et al., 2022).

#### 5.2.4. Cholesterol amount

Changing the amount of cholesterol was studied only in O-NV. Cholesterol has an amphilic nature that increases the rigidity of the bilayer by placing the hydrophilic heads toward the surface, while placing the lipophilic tail toward the core. Increasing the rigidity of the bilayers would allow more entrapment and retard the release of FTN. Moreover, presence of more cholesterol would allow the formation of more novasomes and accumulation of more layers over each other (Abdelbary et al., 2017; Emad Eldeeb et al., 2019; Abdelbari et al., 2021). Also, cholesterol has a free ionizable hydroxyl groups. So, we can conclude that increasing the cholesterol amount has a positive significant effect on EE %, PS and ZP and a negative significant effect on release. Changing the amount of cholesterol showed a non-significant effect on PDI.

#### 5.2.5. Stearic acid amount

Studying the effect of stearic acid (C_17_H_35_COOH) was conducted only in O-NV. Increasing the amount of stearic acid demonstrated a positive significant effect on EE %, PS and ZP, a negative significant effect on release and a non-significant effect on PDI. These results could be explained in terms of the high lipophilicity of stearic acid resulted from its large alkyl chain. Moreover, its high phase transition temperature allows the formation of less leaky vesicles (Ahmad et al., 2019). Furthermore, the high melting point of stearic acid would result in greater viscosity that reduces the efficacy of sonication process regarding PS (Abdelbary et al., 2017). As a general rule the larger the EE %, the larger the PS and the slower the release since the drug would favor the lipophilic vesicles than the release medium (Abdelbary & Aburahma, 2015). Stearic acid also has a free carboxylic group that increases the negative charge on the surface upon ionization.

#### 5.2.6. Ceramide amount

The effect of ceramide amount was examined only in T-CE. Increasing the amount of ceramide resulted in positive significant effect on EE % and PS. However, a negative significant effect on ZP and a non-significant effect on PDI were realized. These findings could be justified regarding the higher lipophilicity and the increased viscosity of the medium upon that prevents the diffusion of FTN to the external medium (Abdelgawad et al., 2017; Yousry et al., 2019). Moreover, ceramide has a low ability to permeate across the membrane leaflet, thus tends to accumulate resulting in large PS (Castro et al., 2014). Accumulation of ceramide tend to hinder the charge on the surface, thus ZP decreases (Yilmaz & Borchert, 2005).

#### 5.2.7. Oleylamine amount

The effect of oleylamine amount was clarified only in O-OLN. Increasing the amount of oleylamine demonstrated a positive significant effect on EE %, ZP and release. However, PS and PDI revealed non-significant effects. The lipophilic properties of oleylamine (C_18_H_37_N) result from its long alkyl chain. Furthermore, it has a strong gelling properties resulted from its ability to create a 3D fibrillar arranges entrapping large amounts of FTN (Bajani et al., 2018; Vizcarra-Pacheco et al., 2021). Moreover, oleylamine has the ability to prevent the aggregation of the adjacent olaminosomes as a strong capping agent via interaction with the carboxylic groups of oleic acid, thus increasing the stability of the formed olaminosomes, thus ZP increases (Javed et al., 2020; Mbewana-Ntshanka et al., 2020). Oleylamine also reduces the interfacial tension with surrounding medium and maintain small PS, thereby release increases.

#### 5.2.8. Terpenes amount

Changing the amount of terpenes was examined in O-TP and V-TP. Increasing the amount of terpenes resulted in negative significant effect on both EE % and PS in O-TP and V-TP. Increasing the amounts of terpenes destabilize the nanosystem by formation of pores, also increase the fluidity of the vesicles, hence EE % decreases (Rangsimawong et al., 2014; Faisal et al., 2018). Reducing PS upon increasing the amount of terpenes could be explained in terms of their ability to minimize the viscosity and interfacial tension, thus preventing Ostwald ripening of the resulted vesicles (Albash et al., 2021). PDI demonstrated a negative significant effect in O-TP resulted from the great association between PS and PDI as they remain minimum with increasing the terpene amount (Kumar et al., 2014). ZP showed a non-significant effect regarding O-TP and V-TP.

#### 5.2.9. Terpenes type

The effect of terpene type was studied only in O-TP (limonene, fenchone and eugenol). Log p values for limonene, fenchone and eugenol were (4.57, 3.52, 2.27 respectively) (Albash et al., 2021). Regarding EE %, the terpene with highest lipophilicity (highest log P) revealed maximum EE % and PS. Limonene has the highest lipophilicity followed by fenchone then eugenol, thereby largest EE % and PS (El-Nabarawi et al., 2018; 2020). Changing terpene type revealed a non-significant effect on PDI.

#### 5.2.10. Sodium deoxycholate amount

Studying the effect sodium deoxycholate (SDC) amount was done only in V-TP. Increasing the amount of SDC significantly decreases EE % and PS. However non-significant effects considering ZP and PDI were realized. As the amount of SDC increases, more micelles would be formed in the dispersion medium. The enhanced solubility of FTN in the micelles would diminish its entrapment inside the vesicles. Moreover, SDC has a fluidizing effect that reduces the rigidity of the bilayers and loss of FTN, thereby EE % decreases (Niu et al., 2014; Ahmed et al., 2020). Regarding PS, the smaller PS could be clarified as a result of reducing the interfacial tension with the surrounding medium and minimizing the aggregation of neighbored particles (Albash et al., 2019).

#### 5.2.11. Ethanol concentration

Examining the consequence of increasing ethanol concentration was achieved only in V-TP. Increasing the amount of ethanol significantly reduces EE % and PS. However non-significant results regarding ZP and PDI were obtained. Reduced EE % and PS could be clarified in terms of increasing the fluidity of the bilayers resulting in depletion of FTN. Furthermore, changing the surface charge of ethanol is accompanied with reducing the stability of the formed vesicles (Imam et al., 2017; Albash et al., 2020).

## 6. Comparisons between the optimum formulae

In order to select the optimum formula of each nanosystem, all the designs were subjected into numerical optimization utilizing Design-Expert^®^ software taking in consideration the highest EE %, ZP (as absolute value) and *in vitro* drug release and smallest PS and PDI. Afterward, comparison between the observed and predicated response to ensure the validity of the optimization process (small % deviation) was done. Table 3 clarifies the values of each response for the optimum formulae. Table 4 shows the conducted *in vitro, ex vivo and in vivo* studies to characterize these formulae. The optimum formulae were compared in terms of the measured responses alongside *in vitro, ex vivo and in vivo* studies. Table 5 provides a detailed summary of the outcomes of all the studied formulae

**Table 3.** Characterizations of the optimum formulae of the studied nanosystems.

Route	Topical		Ocular			Vaginal
Nanosystem	T-T-NV	T-CE	O-NV	O-OLN	O-TP	V-TP
EE %	100.00 ± 1.10	83.00 ± 1.63	94.31 ± 2.75	84.24 ± 1.28	79.02 ± 2.35%	62.18 ± 1.39%
PS (nm)	358.60 ± 10.76	551.60 ± 23.84	197.05 ± 9.97	117.55 ± 5.44	287.25 ± 9.55	310.00 ± 8.16
ZP (absolute -mV)	30.00 ± 0.80	20.90 ± 0.55	66.95 ± 0.92	74.85 ± 1.91	36.15 ± 1.06	10.19 ± 0.2
Release (8h)	< 40 %	not studied	85.33 ± 1.35	77.95 ± 1.22	>80%	not studied

**Abbreviations:** EE %, percent entrapment efficiency; PS, particle size; ZP, zeta potential.

**Nanosystems:** O-NV, Ocular Novasomes; O-OLN, Ocular Olaminosomes; O-TP, Ocular Terpesomes; T-CE, Topical cerosomes; T-T-NV, Topical Trans-Novasomes; V-TP, Vaginal Terpesomes.

**Table 4.** *In vitro, ex vivo and in vivo* studies on the optimum formulae of the studied nanosystems.

Route		Topical		Ocular			Vaginal
Nanosystem		T-T-NV	T-CE	O-NV	O-OLN	O-TP	V-TP
In vitro	Differential scanning calorimetry			+	+		+
	Fourier transform infrared spectroscopy			+	+		
	Transmission electron microscopy	+	+	+	+	+	+
	pH measurement			+	+	+	
	Effect of short-term storage	+	+	+	+	+	
	Effect of gamma sterilization			+	+		
	Minimum Inhibitory Concentration	+		+	+	+	
	Elasticity evaluation						+
	Docking study	+					
	Molecular dynamics simulation	+					
ex vivo	Ex vivo permeation			+	+		+
	Ex vivo corneal hydration			+	+		
in vivo	irritancy test			+	+		
	Histopathology		+	+	+	+	+
	In vivo uptake			+	+		
	Antifungal activity			+	+	+	+
	Kinetic study		+				
	Clinical appraisal	+					

**Nanosystems:** O-NV, Ocular Novasomes; O-OLN, Ocular Olaminosomes; O-TP, Ocular Terpesomes; T-CE, Topical cerosomes; T-T-NV, Topical Trans-Novasomes; V-TP, Vaginal Terpesomes.

**+:** Test was done.

**Table 5.** Main findings of the studied nanosystems.

	*Measured responses*	*In vitro tests*	*Ex vivo tests*	*In vivo tests*
T-T-NV	Very high EE %, acceptable PS, high stability (ZP) and slow release.	spherical vesicles, high stability following storage, effective antifungal activity and high binding stability	–	improved antifungal activity
T-CE	Acceptable EE %, acceptable PS and high stability	fiber-like shape particles and high stability following storage	–	High safety and prolonged residence time
O-NV	High EE %, minute PS, high stability and fast release	Complete entrapment of FTN, spherical vesicles, acceptable pH, high stability following storage or gamma irradiation and effective antifungal activity	enhanced corneal permeation with remarkable corneal safety	High safety, enhanced corneal permeation and improved antifungal activity
O-OLN	Acceptable EE %, minute PS, high stability and fast release	Complete entrapment of FTN, spherical vesicles, acceptable pH, high stability following storage or gamma irradiation and effective antifungal activity	enhanced corneal permeation with remarkable corneal safety	High safety, enhanced corneal permeation and improved antifungal activity
O-TP	Acceptable EE %, small PS, high stability and fast release	spherical vesicles, acceptable pH, high stability following storage and effective antifungal activity	–	High safety and improved antifungal activity
V-TP	Small EE %, acceptable PS and good stability	Complete entrapment of FTN, spherical vesicles and superior elasticity	Improved permeation profile	High safety and improved antifungal activity

**Abbreviations:** EE %, percent entrapment efficiency; FTN, Fenticonazole Nitrate; PS, particle size; ZP, zeta potential.

**Nanosystems:** O-NV, Ocular Novasomes; O-OLN, Ocular Olaminosomes; O-TP, Ocular Terpesomes; T-CE, Topical cerosomes; T-T-NV, Topical Trans-Novasomes; V-TP, Vaginal Terpesomes.

### 6.1. Measured responses

Topical nanosystems include T-T-NV and T-CE. Ocular nanosystems include O-NV, O-OLN and O-TP. Vaginal nanosystem include only V-TP. Regarding topical nanosystems, T-T-NV revealed significantly (*p* < 0.05) larger EE % and ZP (absolute value) in addition to smaller PS. So, the optimum formula of T-T-NV is expected to be more efficient than the optimum formula of T-CE. Considering ocular nanosystems, O-NV had significantly (*p* < 0.05) larger EE % than O-OLN and O-TP. Furthermore, O-OLN had significantly (*p* < 0.05) smaller PS and larger ZP than O-NV and O-TP. However, both O-NV and O-OLN showed PS < 200 nm that provides more efficient drug permeation through the ocular tissue. Also both O-NV and O-OLN showed high ZP (>30 mV) that ensures the stability of the two nanosystems (Muller et al., 2001). Considering the amount of FTN released after 8 h, O-NV demonstrated significantly (*p* < 0.05) higher release (> 80%) than O-OLN. Thereby, O-NV is expected to be the most efficient ocular nanosystem.

The best topical (T-T-NV) and ocular (O-NV) formulae were compared together with the vaginal nanosystem (V-TP) to predict the best nanosystem. O-NV showed significantly (*p* < 0.05) larger ZP and smaller PS than T-T-NV and V-TP. Regarding EE %, O-NV revealed significantly (*p* < 0.05) higher EE % than V-TP. Although T-T-NV had the highest EE %, it showed a non-significant (*p* > 0.05) difference with O-NV. Moreover, O-NV revealed release > 80% after 8 h. From the previous results, we can predict that O-NV would be the best nanosystem.

### 6.2. In vitro characterizations

#### 6.2.1. Differential scanning calorimetry (DSC)

**Figure 1.**
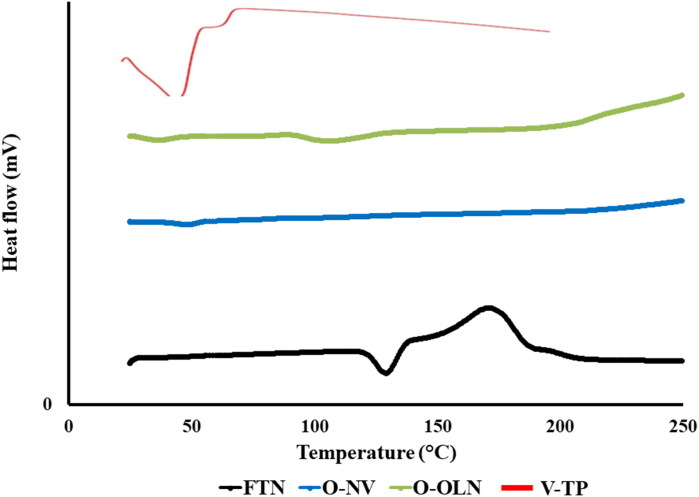
DSC thermograms of pure Fenticonazole (FTN), O-NV, O-OLN and V-TP (Albash et al., 2020; Ahmed et al., 2022a,b).

DSC was conducted for O-NV, O-OLN and V-TP. DSC test is generally performed to ensure the complete entrapment of FTN (disappearance of FTN characteristic peak). Moreover, disappearance of FTN peak would demonstrate its existence in amorphous state (Albash et al., 2019). DSC also gives an indication for interaction between different components. In order to conduct DSC test, a certain weights of the optimum formulae or FTN were subjected to a gradual heating process under a nitrogen steam to obtain peaks that would provide an indication of entrapment. FTN had a characteristic endothermic peak at 135 °C which is connected to its melting point (Kim et al., 1989; Albash et al., 2020). Figure 1 reveals the DSC thermogram for O-NV, O-OLN and V-TP. All the nanosystems demonstrated complete entrapment of FTN (Albash et al., 2020; Ahmed et al., 2022a,b).

#### 6.2.2. Fourier transform infrared spectroscopy (FTIR)

**Figure 2.**
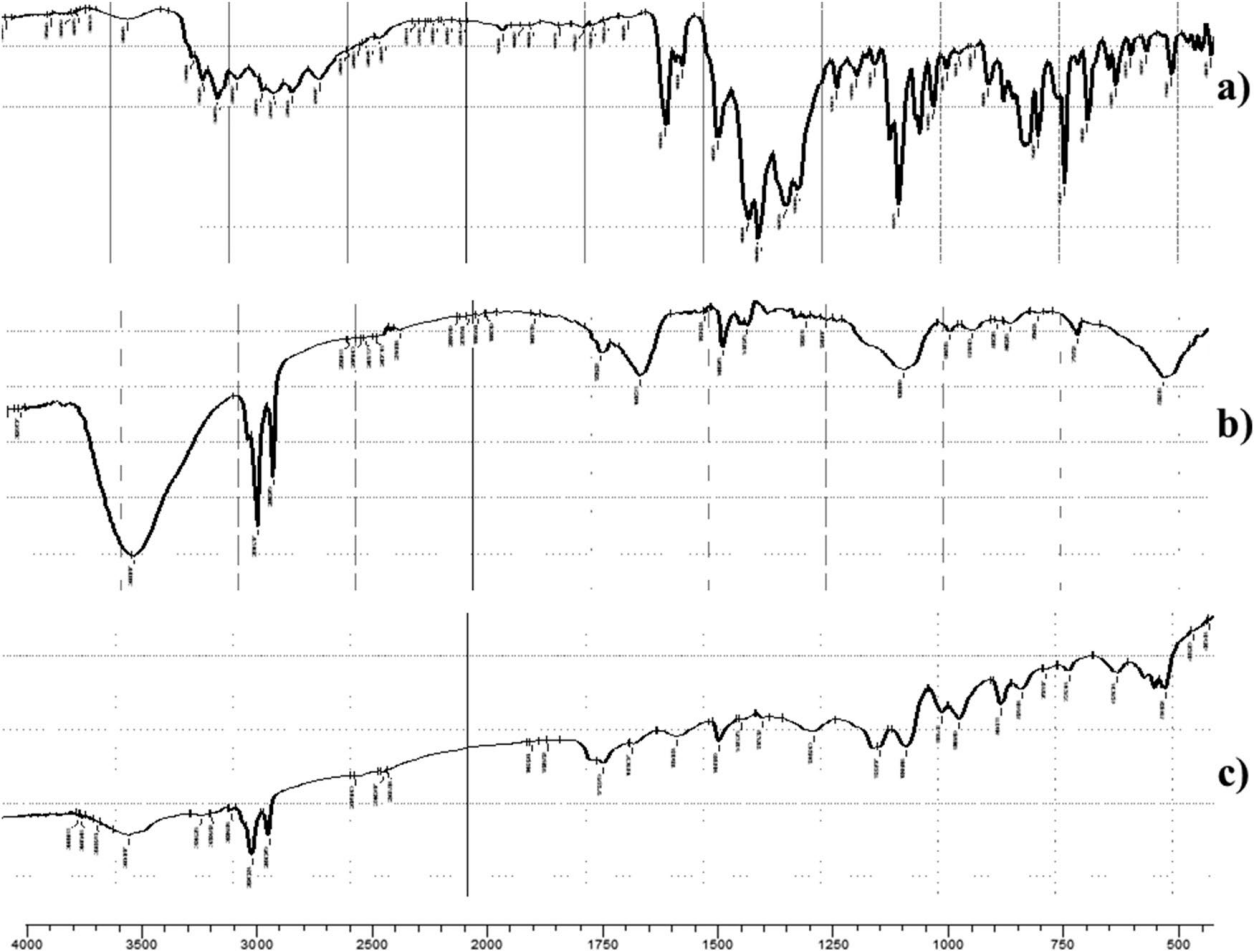
FTIR spectra of (a) pure Fenticonazole Nitrate (b) O-NV (c) O-OLN (Ahmed et al., 2022a,b).

FTIR was studied in O-NV and O-OLN. FTIR confirms the entrapment of FTN by the disappearance of its characteristic peaks. Moreover, FTIR could be used to detect any possible interaction between the components (Sayed et al., 2018; Ahmed et al., 2020). FTIR was done by mixing a certain weight of the optimum formulae or FTN with dry potassium bromide and squeezed into disk. the resulted disk was studied in the range of 4000 − 500 cm^−1^ at 25 ± 2 °C using FTIR spectrophotometer (model 22, Bruker, Coventry, UK). The FTIR results for both O-NV and O-OLN came in harmony with DSC as the peaks of FTN vanished (Ahmed et al., 2022a,b). Figure 2 shows the FTIR for both O-NV and O-OLN. For O-OLN the interaction between carboxylic group oleic acid and amino group of oleylamine was confirmed through the appearance of amide bond at 3332.99 cm^−1^ (Amide A peak) and a stake-shaped band close to 1710 cm^−1^ for the C = O stretch (Amide I peak) (Durukan et al., 2019; Ji et al., 2020).

#### 6.2.3. Transmission electron microscopy (TEM)

**Figure 3.**
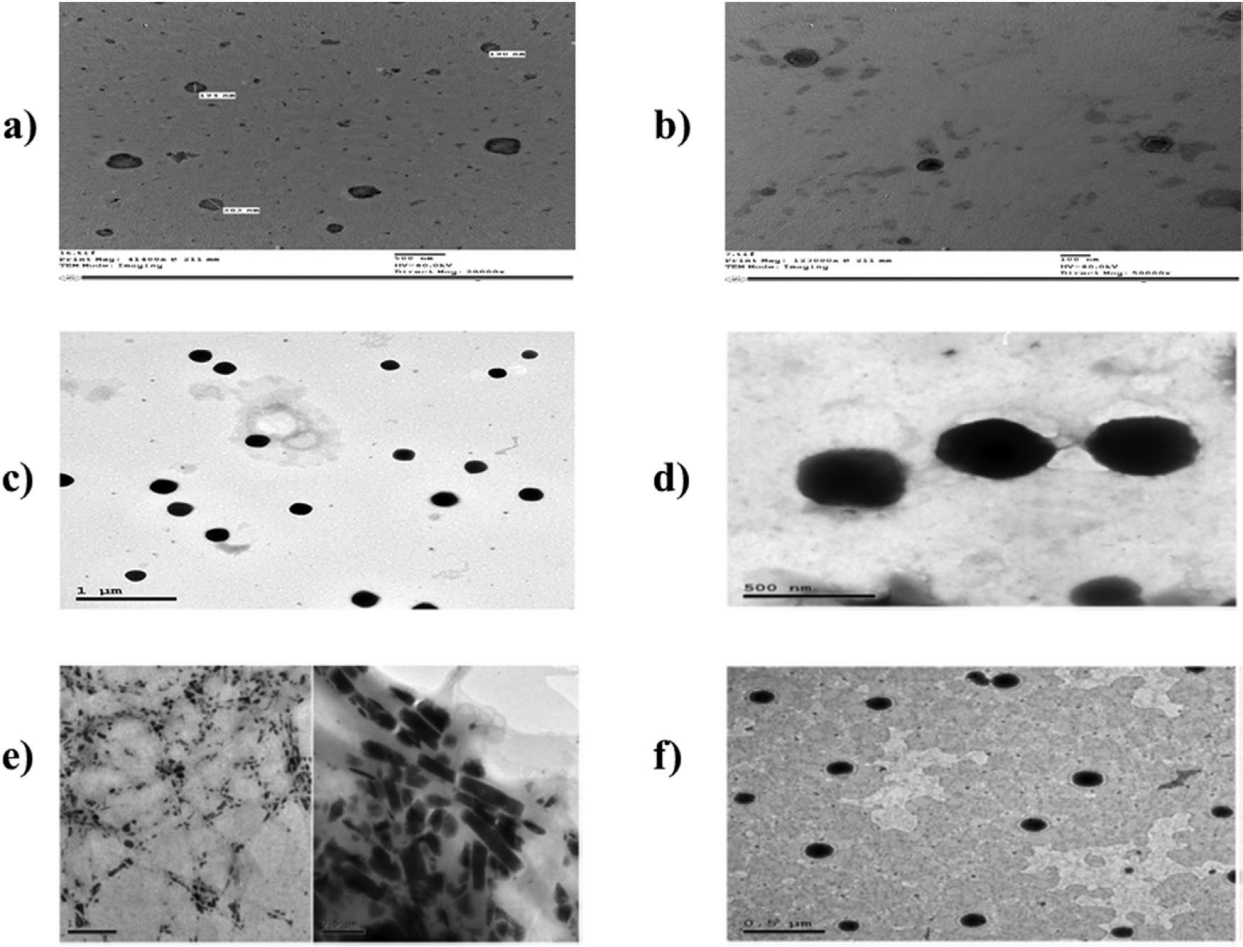
TEM images of (a) O-NV (b) O-OLN (c) O-TP (d) T-T-NV (e) T-CE (f) V-TP (Albash et al., 2020; 2021a; 2021b; Ahmed et al., 2022a; 2022b; Albash et al., 2022).

TEM provides a confirmation for the results of PS obtained by Zetasizer. TEM was performed for all the studied nanosystems. At the beginning, the optimum formulae were diluted to suitable concentration. Afterward, sited over carbon coated copper rods, dehydrated at 25 ± 2 °C and then, stained by phosphotungstic acid. O-NV, O-OLN, O-TP, T-T-NV and V-TP showed spherical shape particles. However, T-CE revealed fiber-like shape particles. Figure 3 reveals TEM pictures for the studied formulae.

#### 6.2.4. pH measurement

Measurement of pH is important to ensure the suitability of the administrated formula to body tissue. pH may also affect the permeation of the administrated nanosystem (Mohanty et al., 2013). This test was performed for the all ocular nanosystems since the eye is a very delicate organ. pH was determined using a pH meter without any dilution. Generally, pH of regular ocular preparations fluctuated between 3.50 and 8.50 (Said et al., 2021). O-NV, O-OLN and O-TP demonstrated a suitable ocular pH that ensured the safety of these formulae.

#### 6.2.5. Effect of short-term storage

This study is conducted to confirm the ability of nanosystems to maintain their efficacy and characters following the preservation under a selected environment. All optimum formulae except V-TP were stored at (4–8 °C) for nearly three months (Ahmed et al., 2023). Afterward, the stored formulae were compared to the fresh formulae regarding the previously measured responses (EE %, PS, ZP and release). Moreover, their physical appearance were reevaluated to confirm the absence of any aggregates (Al-Mahallawi et al., 2017; Fahmy et al., 2021). O-NV and O-OLN also studied the similarity between the release profiles before and after the storage employing the Similarity factor “ƒ_2_” equation (Sayed et al., 2020; 2021). R_t_ and T_t_ represent % FTN released from the fresh and preserved formula respectively at time t. The value of ƒ2 must be between 50 and 100 to ensure the similarity (Abdelbary et al., 2016). All the preserved formulae demonstrated the absence of aggregates alongside maintaining their measured responses (*p* > 0.05). O-NV and O-OLN revealed ƒ2 > 50, thereby similar release profiles.

(Eq. 4) $$f_{2}=50.\log\{[1+(\frac{1}{n})\sum_{t=1}^{n}{\left(R_{t}-T_{t}\right)}^{2}{]}^{-0.5}.100$$

#### 6.2.6. Effect of gamma sterilization

This test is performed to ensure the ability of nanosystem to preserve its physical properties, measured responses and release profile following the sterilization process by gamma radiations. Gamma sterilization was performed in presence of dry ice to prevent any undesirable consequences. Cobalt-60 irradiator at rate of 1.774 kGy/h was employed with a radiation dose of 25 kGy in an Indian Gamma cell (Ahmed et al., 2022). Afterward, the resulted formulae were compared to the freshly prepared optimum formulae as previously discussed under effect of storage. Sterilization is important to eradicate any microbial contamination that could infect the body tissue especially the eye (Sayed et al., 2020). This test was performed for O-NV and O-OLN and both of them verified the absence of aggregates, unchanged responses (*p* > 0.05) and similar release profiles (ƒ2 > 50) (Ahmed et al., 2022a,b).

#### 6.2.7. Minimum inhibitory concentration (MIC) determination

Broth Microdilution Technique was used in MIC determination with respect to the Clinical and Laboratory Standards Institute guidelines (Humphries et al., 2018). Briefly, 150 μL of two-fold strength Sabouraud dextrose broth (SDB) was added to each well of a sterile U-shaped bottom 96-well plate. An equal volume of the tested formulae was added only to the first well of each row. The wells were then inoculated with 10 μL of *Candida albicans* ATCC 60193 suspension (10^7^ CFU/mL). A negative control for sterility (neither yeast nor tested formula was placed) and another well as a positive control for growth (inoculated with yeast suspension only) were placed in each row. Incubation was done at 25 ± 2 °C for 24 hours in aerobic environment. MIC was the lowest concentration without any noticeable microbial growth (Albash et al., 2021). MIC test was conducted to determine the lowest concentration with effective antimicrobial properties (Albash et al., 2021; Fahmy et al., 2021). MIC was performed for T-T-NV, O-NV, O-OLN and O-TP. All these formulae showed a significantly (*p < 0.05*) lower MIC than FTN suspension resulted from the small PS, the high ZP and enhanced permeation properties of the constructed formula (Ing et al., 2012; Fahmy et al., 2021).

#### 6.2.8. Elasticity evaluation

This test is done to confirm the ability of the administrated nanosystem to penetrate the mucus membrane. Elasticity was measured for V-TP only. For effective vaginal treatment, the nanosystem should pass through the luminal and adherent mucus layers then reach the underlying epithelium (Wong et al., 2014). Extrusion technique was used to determine the elasticity of the studied formula. Briefly, a certain pressure was applied to force the vesicles to pass through a filter with a diameter of 200 nm. The elasticity was defined in terms of percentage change (%) in the size of the vesicles after extrusion. The optimum V-TP revealed superior elasticity due to the synergistic effect of both ethanol and terpene as membrane softeners (Subongkot et al., 2012; Albash et al., 2020).

#### 6.2.9. Docking study

This test is performed to determine the possible interaction between FTN and the different components since each component will act as receptor for FTN. This test was only conducted in T-T-NV. Molecular operating environment (MOE) software version 2019.0102 was used in the docking evaluations. Firstly, ChemDraw 20.1.1 was used to sketch the studied compounds followed by 3D conversions utilizing MOE. All the docking studies were performed at the highest accuracy utilizing induced fit protocol and triangle matcher as a scoring function. Both docking scores and molecular interactions were used in docking analysis. This test highlighted the importance of cholesterol for the formation of the trans-novasomes as it behaves as a carrier for FTN (Albash et al., 2022).

#### 6.2.10. Molecular dynamics simulation (MDS)

MDS ensures the binding stability between the ligand and receptor with superior techniques based on millions of conformations (Alamri et al., 2021). Both MOE and NAMD 2.1.1 software were used in MDS evaluations. The root means square deviation (RMSD) of all heavy atoms in the system was determined utilizing the observed trajectories from the production phase. MDS was only conducted in T-T-NV with a demonstration of the high binding stability between FTN and cholesterol. These results came in harmony with the previously discussed docking study (Albash et al., 2022).

### 6.3. Ex vivo characterizations

#### 6.3.1. Ex vivo permeation

**Figure 4.**
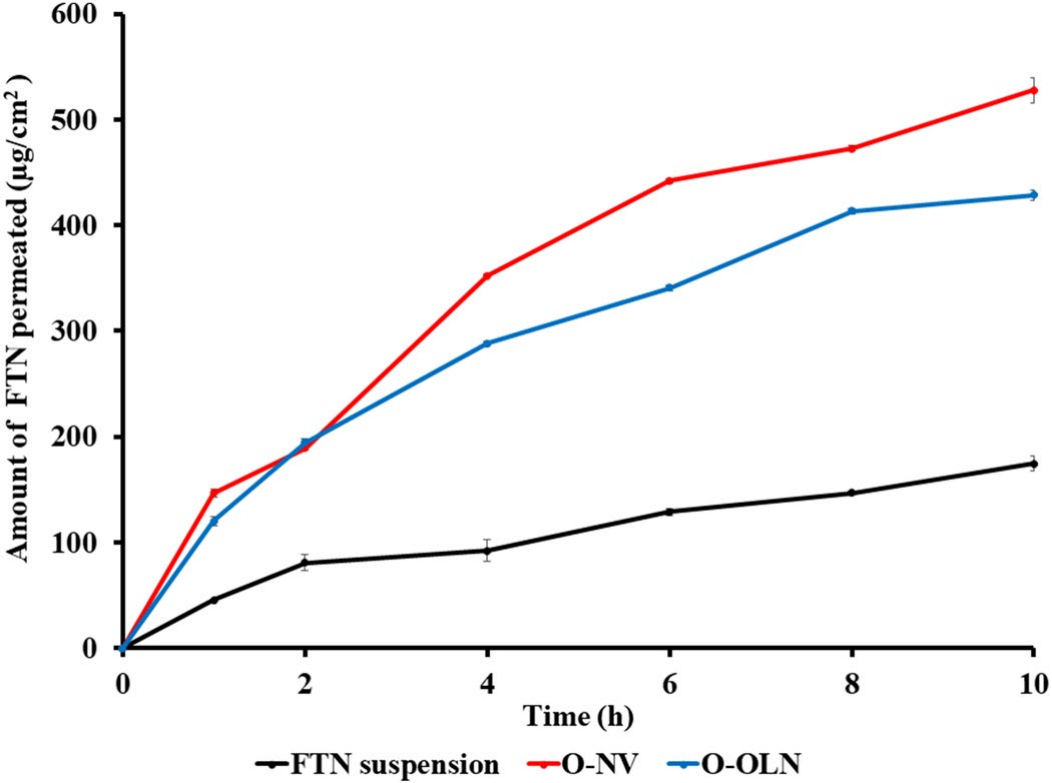
*Ex-vivo* permeation profiles of O-NV and O-OLN compared to FTN suspension at 37 ± 0.5 °C (Ahmed et al., 2022a,b).

*Ex vivo* permeation study is conducted to recognize the permeation properties of the optimum formula. O-NV and O-OLN were subjected to this test on freshly separated rabbit corneas (Ahmed et al., 2022a,b). The corneas were attached to one end of the open ended tube. Certain volume of the optimum formulae was placed in the donor medium. Moreover, certain volume of phosphate buffer saline solution (pH 7.4) containing 25% ethanol was placed in the receptor medium. At a defined time-intervals, aliquots were withdrawn, clarified using 0.45 μm membrane filter then evaluated by HPLC (Shimadzu, Tokyo, Japan) (Ahmed et al., 2022). Figure 4 shows the permeation profile of the optimum formulae of O-NV and O-OLN compared to FTN suspension. Both O-NV and O-OLN revealed significantly (*p < 0.05*) higher permeation than FTN suspension. These results could be explained in terms of the small PS that would extend residence time and assists the movement across the hydrated network of the corneal stroma (Younes et al., 2018). Furthermore, the softening effect of surfactants and the high ZP (Li et al., 2014). For O-NV the presence of cholesterol facilitates the permeation of the optimum formula as permeation enhance. Moreover, stearic acid leads to lipid instability as a result of increasing the curvature stress (Singh et al., 2020). Regarding O-OLN, capping effect of oleylamine and permeation enhancer properties of oleic acid had an added value (Manconi et al., 2009; Abdulbaqi et al., 2016). O-NV had a significantly (*p < 0.05*) higher permeation after 10 h compared to O-OLN. These results came in harmony with the previously expected higher efficacy of O-ON than O-OLN.

Regarding V-TP, the permeation study was performed using a freshly harvested vaginal tube (Albash et al., 2020). This study was performed using locally fabricated Franz’s diffusion cell. phosphate buffer saline solution (pH 4.5) containing 25% ethanol was used as a receptor medium. The obtained samples were analyzed by a validated HPLC method (Albash et al., 2020). V-TP demonstrated higher permeation profile compared to FTN suspension (*p < 0.05*). The high permeation properties of V-TP resulted from its small PS together with the enhanced fluidity caused by terpene and alcohol.

#### 6.3.2. Ex vivo corneal hydration

The corneal hydration level (HL%) was determined in both O-NV and O-OLN to detect any possible corneal injury after the *ex vivo* permeation study. Briefly, the used corneas in *ex vivo* permeation study were delicately washed to get rid of any remained formula, gently wiped to remove the excess water then weight before (wet cornea weight) and after (dry cornea weight) dehydration at 50 °C for 24 h. Generally, for normal healthy cornea the HL % should lie between (76–80%) (Huang et al., 2017). Both O-NV and O-OLN formulae had an HL% within the range. Moreover, a non-significant (*p > 0.05*) difference with FTN-suspension was recognized, thereby these formulae could be considered safe. These findings validate the previously mentioned results regarding safety. O-TP needs more studies regarding *ex vivo* corneal permeation and *ex vivo* corneal hydration to confirm its corneal efficacy and safety alongside the *in vivo* tests. HL % was calculated using the following equation (Ahmed et al., 2022):

(Eq. 5) $$HL\;\%=[1-(\frac{W_{d}}{W_{w}})].100$$

### 6.4. In vivo characterizations

#### 6.4.1. Ocular irritancy test

This test was performed to confirm the safety of the optimum formulae O-NV and O-OLN. It was conducted by single administration of the studied formulae into one eye, the other eye would function as a control. Both eyes were carefully examined for any sign of inflammation by direct visual inspection using a slit lam for 24h. Both formulae were considered safe as they didn’t show any sign of redness (Abdelbary et al., 2017). O-TP didn’t conduct the ocular irritancy test.

#### 6.4.2. Histopathological study

**Figure 5.**
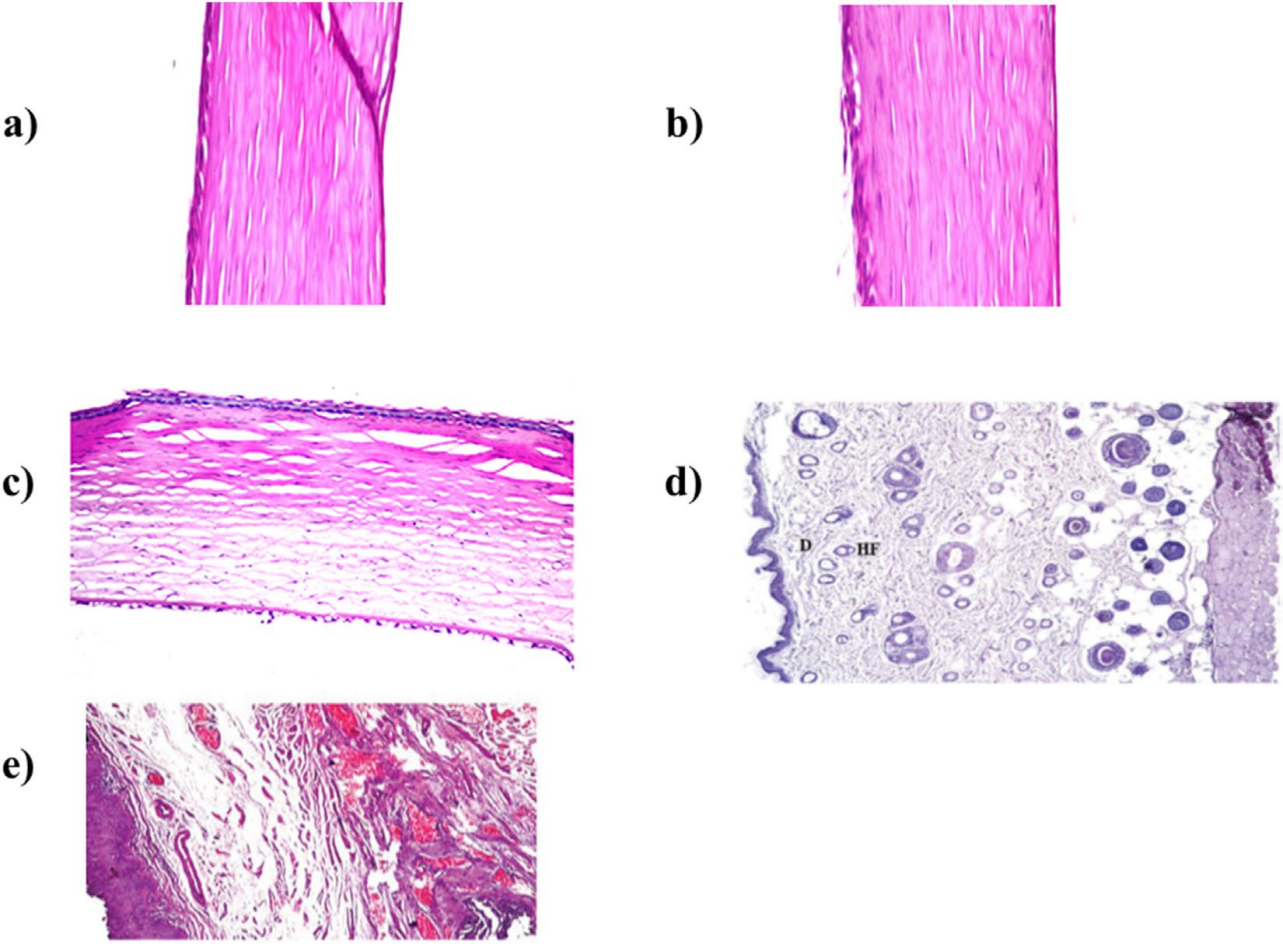
Histopathological photomicrographs after instillation of a) O-NV (rabbit cornea), b) O-OLN (rabbit cornea), c) O-TP (rabbit cornea), d) T-CE (rat skin) and e) V-TP (vaginal tissue) (Albash et al., 2020; 2021a,b; Ahmed et al., 2022a,b).

A histopathological study is generally conducted to recognize any tissue injury resulted from optimum formula. The histopathological studies of ocular nanosystems O-NN, O-OLN and O-TP were conducted using rabbits’ corneas. In brief, the optimum formulae were compared to sterile normal saline (negative control) and isopropyl alcohol 95% (positive control). The optimum formulae were instilled into one eye, while the other received either negative or positive control. Administration was done twice daily for one week prior to animal scarification. The separated corneas were washed with tap water then serial dilutions of alcohol (methyl, ethyl and absolute ethyl) for dehydration. They were then treated with xylene and embedded in paraffin at 56 °C for 24 h in hot air oven. The resulted tissue sections were collected on glass slides, deparaffinized, stained by hematoxylin & eosin stain for assessment through the light electric microscope. For topical T-CE, rat skin was used. Regarding V-TP, vaginal tissue was used. Figure 5 represents the histopathological results of the studied formulae. All the studied formulae didn’t reveal any histological change, thereby considered safe.

#### 6.4.3. *In vivo* uptake

**Figure 6.**
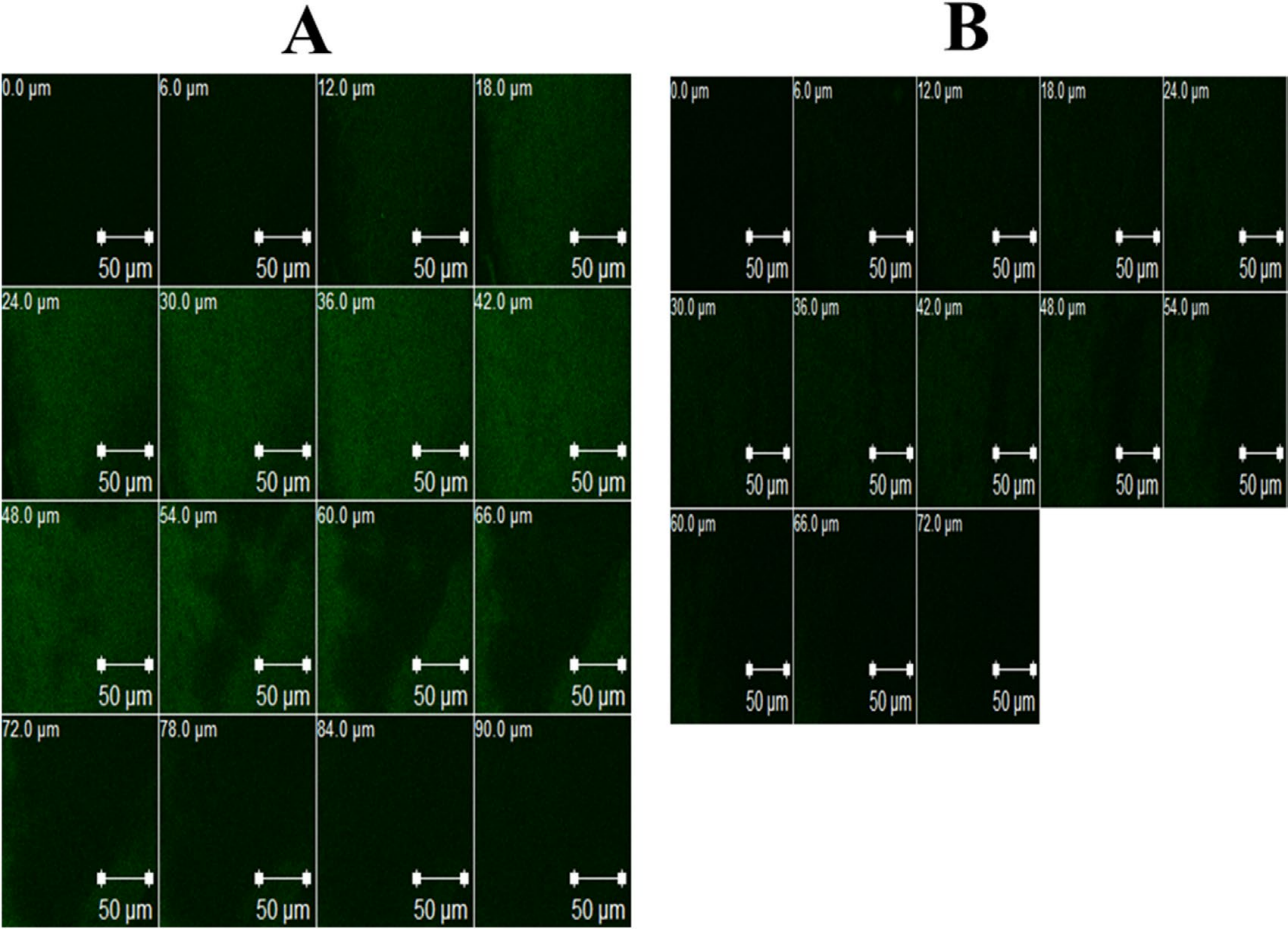
Confocal laser scanning micrographs (CLSM) of rabbits’ corneas after instillation of:. a) RhB-loaded O-NV and b) RhB-loaded O-OLN (Ahmed et al., 2022a,b).

This test validates the permeation properties of nanosystem in a living tissue. Generally, the drug (FTN) is replaced by 0.1% w/w Rhodamine B (RhB) and visualized using Confocal laser scanning microscopy (CLSM) (Elsayed & Sayed, 2017). Briefly, one drop of RhB-loaded formula (100 µL) was administrated into the right eye male albino rabbit and the other eye serve as control since it received RhB-loaded aqueous solution. Animals were sacrificed after 6 h to obtain the studied corneas which were imaged at the same day at 485 and 595 nm using argon and helium–neon lasers, respectively. LSM software version 4.2 (Carl Zeiss Microimaging, Jena, Germany) was used to process the resulted images. *In vivo* uptake test was performed by O-NV and O-OLN only. O-NV revealed deeper permeation (90 μm) than O-OLN (72 μm), as shown in Figure 6. This matches with the results of the earlier *ex vivo* permeation study. Ability of nanosystem to carry drug into deeper corneal tissue offers a significant solution for deep fungal infections (Younes et al., 2018).

#### 6.4.4. Antifungal activity

For the ocular nanosystems O-NV, O-OLN and O-TP, susceptibility test was performed. Briefly rabbits were divide into two groups (FTN suspension and the optimum formula). The tested organism was *Candida albicans* ATCC 60193. The lower conjunctival sac of the right eye of each rabbit received FTN suspension or the optimum formula using a micropipette, while the left eye of each rabbit served as a control. At certain time intervals filter paper disks (Whatman no. 5, 6 mm in diameter) were wetted by placing them under the eyelid of each eye of each rabbit. For each eye (right and left), some disks were placed in an Eppendorf tube (1.5 mL) containing 500 μL Sabouraud dextrose broth (SDB) inoculated with 10% v/v *Candida* suspension (10^7^ CFU/mL). The other disks were put in another Eppendorf tube containing 500 μL uninoculated SDB; this was used as a blank during measuring the optical densities. All the Eppendorf tubes were then incubated at 25 ± 2 °C for 24 hours. Susceptibility test records the % Growth inhibition. All the formulae revealed the maximum % Growth inhibition two-hours post-administration. All the optimum formulae revealed significant (*p* < 0.05) increase in maximum % Growth inhibition when compared to FTN-suspension. O-TP had the highest % Growth inhibition two-hours post-administration. However, O-TP demonstrated significant (*p* < 0.05) difference when compared to O-OLN and non-significant (*p* > 0.05) difference was detected when compared to O-NV. We can conclude that O-NV is the best ocular nanosystem as it was subjected to extensive *ex vivo* and *in vivo* tests with superior results when compared to O-OLN or O-TP.

For assessing the antifungal activity in the vagina, rats were used. Candidiasis was firstly induced by intravaginal injection of candida albicans cultures dissolved in 0.10 µL of saline by automatic micropipette, the control rats were inoculated intravaginally with saline. All the rats were left for 4 days with regular inspection to ensure the absence of weight loss or toxicity following the injection. After that, treatment started and continued for a week by administrating 20 µL from the treatment by micropipette. Some rats served as control and were kept untreated. The vaginal nanosystem V-TP showed significant (*p* < 0.05) lower concentration of *Candida albicans* than FTN-gel.

#### 6.4.5. Kinetic study

The study was done only by T-CE to determine the deposited amount of FTN on the skin at different time intervals. Animals were divided into two groups receiving either the optimum formula or FTN suspension. Animals were sacrificed at scheduled time intervals and the removed skins were cut into small pieces and sonicated with methanol. The extract was then filtered using 0.45 μm membrane filter then evaluated by a validated HPLC method. The optimum formula revealed superior concentration compared to FTN suspension which is expected to be related to the Brij and ceramide content of T-CE. Brij has the ability to prolong the residence time via modulation of the interfacial properties of the nanosystem (Vega et al., 2013). However, ceramide enhanced the permeation properties as it could interact with the keratin of the corneocytes (Abdelgawad et al., 2017).

#### 6.4.6. Clinical appraisal

T-T-NV was used clinically on patients suffering from tinea corporis and without a significant difference between them. Patients were divided into two groups receiving either the optimum formula or the marketed Miconaz® cream without any other simultaneous use of topical, systemic antifungal, systemic antihistamine, or systemic corticosteroid agent. Photographs of lesions were taken before treatment and after 4 weeks or complete improvement. For mycological examination, skin scrapings were collected then treated with 20% potassium hydroxide (KOH) on a glass slide prior to its microscopic examination. The optimum formula revealed a higher antifungal activity compared to the marketed Miconaz® cream within 4 weeks. T-T-NV is the only formula that was tested on human patients (Albash et al., 2022).

## 7. Future perspectives

All the formulae except T-T-NV hadn’t undergo any clinical studies. Moreover, the clinical study in T-T-NV was done on only 40 patients. The previous *ex vivo* or *in vivo* tests rely mainly on animals which won’t be enough for marketing production. So, more detailed *in vivo* studies are mandatory for the best optimum formulae of each route (T-T-NV, O-NV and V-TP). Pharmacokinetic studies hadn’t received a comprehensive discussion in the studied nanosystems. Also, we conclude that O-NV could be the best formula, however we need more specific studies regarding each route to emphasize the activity of O-NV throughout the body. Furthermore, recent nanosystems enclosing FTN haven’t discussed some other route of administration such as oral, aerosol, nasal sprays and injection. Future nanosystems could be utilized to achieve superior safety and efficacy such as smart nano-platforms that could significantly alter their mechanical, thermal, and/or optical properties in a manageable or expectable means. Finally, extracellular vesicles (exosomes) that could start different physiological and pathological consequences could be studied.

## 8. Conclusions

All the studied nanosystems were successfully fabricated either by ethanol injection or thin film hydration technique. Many factors could influence the properties of the optimum formula, thereby many statistical designs followed by numerical optimization were done in order to select the best combination of these components. The optimum formulae were compared in terms of the measures responses, *in vitro, ex vivo* and *in vivo* assessments utilizing 95% CI method to detect the significance difference. Regarding topical nanosystems, T-T-NV showed significantly (*p* < 0.05) larger EE % and ZP (absolute value) in addition to smaller PS than T-CE, thereby T-T-NV is expected to be more efficient. T-T-NV showed spherical particles, however T-CE showed fiber-like shape particles and both of them had high stability. Efficacy of T-T-NV was confirmed through MIC determination and clinical appraisal study. More studies are required to ensure the safety of T-T-NV. Safety and efficacy of T-CE was confirmed through histopathological and kinetic studies. Regarding ocular nanosystems, O-NV had significantly (*p* < 0.05) larger EE % and drug release than O-OLN and O-TP, in addition to acceptable PS and ZP. Spherical particles and high stability were confirmed for O-NV, O-OLN and O-TP. Safety of all ocular nanosystems was confirmed through pH measurement and histopathological studies. Their efficacy was confirmed through MIC determination and susceptibility test. O-NV showed superior activity that augment the expected higher efficacy of O-NV. The vaginal V-TP showed spherical particles with high elasticity, permeability and activity. O-NV showed significantly (*p* < 0.05) larger ZP and smaller PS than T-T-NV and V-TP. Moreover, acceptable EE % and release > 80% after 8 h, thus expected to be the best nanosystem.
